# Selection of reference gene in *Eucalyptus camaldulensis* for real-time qRT-PCR

**DOI:** 10.1186/1753-6561-5-S7-P125

**Published:** 2011-09-13

**Authors:** Boby Unnikrishnan, Gurumurthy Demlapura Shankaranarayan, Navin Sharma

**Affiliations:** 1 Division of Plant Molecular Biology, ITC R&D Centre, Bangalore 560058, India

## Background

Real time quantitative reverse transcription PCR (real-time qRT-PCR) is an established technique for quantification of mRNA and has been extensively used for gene regulation studies in plants. However, there are inherent challenges associated with the technique such as variability of RNA and extraction protocols, different rates of reverse transcription and PCR efficiencies. This demands an accurate method of normalization to obtain reproducible results. Among the various methods, normalization to a reference house keeping gene is the most commonly used method. If inappropriate reference genes are used for normalization, the experimental results can vary significantly leading to false results [1].

*Eucalyptus camaldulensis* Dehnh. is a widely distributed tree species used for planting in arid and semi-arid areas. The wood is composed of mainly cellulose and lignin and the pathway involved in lignin formation is fairly understood. The lignin biosynthetic genes*viz Ferulate 5 Hydroxylase* (*F5H*), *4 Coumarate CoA Ligase* (*4CL*), *Cinnamoyl CoA Reductase* (*CCR*) and *Cinnamoyl Alcohol Dehydrogenase* (*CAD*) are highly conserved across the tree species and have been utilized as targets for manipulating lignin content. The present study describes validation of a reference gene in various tissue types of eucalyptus and its subsequent use for the expression analysis of lignin biosynthetic genes.

## Materials and methods

**Figure 1:**
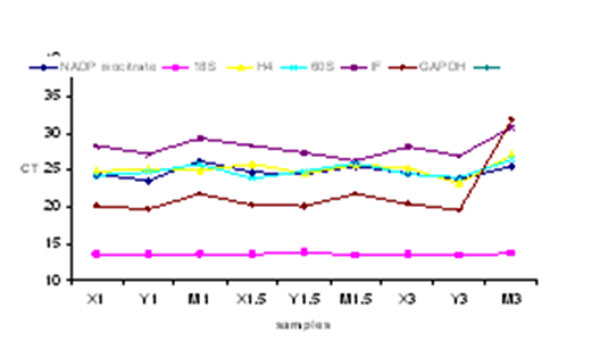
Distribution of average C_T_ values of various house keeping genes under study

Samples were collected from three different tissues of *E. camaldulensis* namely xylem, young leaf and mature leaves and three developmental stages *viz* one, one and half and three years. The samples were named as X (1-3) for all the xylem samples coming from three developmental stages and Y(1-3) and M(1-3) for young leaves and mature leaves respectively (Figure 1). Total RNA was extracted with RNAqueous Kit (Ambion, Austin TX, USA) following manufactures protocol. cDNA was synthesized from 1 μg of RNA using High Capacity cDNA Reverse Transcription Kit (Applied Biosystems, Foster City, CA, USA) with random hexamer primers. Primers for house keeping genes and lignin biosynthetic genes were designed based on the EST data from Eucalyptus using Primer Express 3.0 Software (Applied Biosystems). The nucleotide sequence of *CAD*, *CCR*, *4CL* and*F5H* from *E. camaldulensis* were deposited in NCBI database (HR309064, HR309065, HR309066, HR309067). The real time PCR efficiency of each primer pair was determined with the slope of a linear regression model and all PCRs displayed efficiencies between 90 and 95%. Comparative C_T_ method was followed for quantification of lignin biosynthetic genes with 18S rRNAas the internal control as suggested by Schmittgen and Livak [2]. The stability analysis of each reference gene was carried out by the method given by Brunner *et al*[3].

## Results

The choice of internal control is usually straight forward especially for analysis of samples from same tissues. However, when samples are derived from different tissues or developmental ages, as it is seen in our study, required validated reference gene/ genes. Six different housekeeping genes were chosen for the analysis which included NADP isocitrate dehydrogenase, 18S ribosomal RNA, Histone H4, 60S ribosomal protein L7, Translational initiation factor (TIF) and Glyceraldehyde 3 phosphate dehydrogenase. Among the genes used in the study, 18S rRNAshowed the least C_T_ indicating higher abundance while the highest C_T_ was observed for TIF. Further, 18S rRNA showed maximum stability when C_T_ was plotted against samples (Figure 1). A gene with low stability index is considered to have highest stability [3]. In the present study with E. camaldulensis, 18S rRNA had the lowest stability index while GAPDH showed maximum variation among the samples.

Expression analysis of four lignin biosynthetic genes *viz CCR*, *CAD*, *F5H* and *4CL* were carried out in different samples by using 18S rRNA as the reference gene. Lignin analysis of samples showed higher lignin content in three-year-old samples estimated by klason lignin analysis compared to one-year old. All genes under study showed increased expression in three year old samples except for *CAD* gene. The results also showed increased amount of *F5H* transcripts in older tissues indicating higher syringyl units in older tissue. Chen *et al* .[4] reported syringyl type lignin content and S/G ratio increased from younger internode to older internode while G and H decreased in parallel.

As the expression profile showed variations in different age samples, further experiment was under taken to evaluate the expression levels of these genes in same age trees (Three year old) in correlation with lignin content. Based on klason lignin content, trees with lignin content of 26± 0.3 and 24± 0.2 % were chosen for the studies. All genes under study *viz4CL*, *CAD*, *CCR* and *F5H* showed increase in expression in high lignin sample in comparison with low lignin.

## Conclusions

Among the house keeping genes under study, 18SrRNA exhibited lowest stability index and we are suggesting it as a reference gene for expression studies in Eucalyptus. The reduction in *CAD *transcript level and higher levels of *F5H* in older tissues indicated a possible shift towards an increased syringyl lignin where as when analysis was done at same age, expression levels of all selected lignin biosynthetic genes were high in high lignin tree. The study opens up the possibility for using these genes as candidate genes for the selection of desired genotypes.
